# Audit to Determine the Incidence of Did Not Attend (DNA) Rates at First Assessment in the NHS Northern Gambling Service (NGS) by Assessment Modality

**DOI:** 10.1192/bjo.2024.348

**Published:** 2024-08-01

**Authors:** John Barker, Benjamin Marriott

**Affiliations:** NHS Northern Gambling Service, Leeds, United Kingdom

## Abstract

**Aims:**

Non-attended appointments can lead to adverse outcomes for a service and its users, including reduced service efficiency; increased waiting times; and impaired patient care. The audit objective was to explore whether DNA rates vary between the current modalities of face-to-face; virtual; and telephone. It was hoped that this would enable the service to better understand the reasons for patients not attending initial assessments and determine whether the modality may present a barrier.

**Methods:**

A sample was obtained including all first assessment appointments between March 2022 and March 2023 (n = 386). Data included the modality for each initial appointment. Matched to this data, was whether the patient attended each appointment, creating a frequency of DNAs for each appointment modality across the year. Data analysis was conducted using Microsoft® Excel®. Beyond frequency and percentages, a chi-square test was used to assess for a statistical difference in appointment attendance between modalities.

**Results:**

For this one-year sample the overall attendance rate was 77%: with 299 appointments attended, and 87 ‘DNAs’. The DNA rates across the one-year sample were face-to-face (24%); virtual (22%); and telephone (23%).

The chi-square value produced when analysing the DNA rates between modalities was 0.92 (critical value 5.99). Hence, there was no statistically significant difference in DNA rates by modality.

**Conclusion:**

Despite the absence of variation in DNA rates between modalities, the findings can be viewed as reassuring. The move to include multimedia alternatives to assessments does not appear to be impacting attendance when compared with assessments that continue to occur face-to-face. Balanced against this increased geographical inclusion afforded by remote appointments, is the competing equity issue of digital exclusion, highlighting the need for face-to-face appointment provision to remain accessible across the service.

This audit did not collect demographic data that may have provided insight into whether certain factors may have impacted attendance and could have acted as confounders, for example geographical location.

Introduction of a supportive reminder letter for patients, to bridge the wait between patient's referral and their initial assessment, was an outcome recommendation that was implemented by the service.

